# A Genome-Wide Analysis Indicates that Yeast Pre-mRNA Splicing Is Predominantly Posttranscriptional

**DOI:** 10.1016/j.molcel.2024.07.031

**Published:** 2024-08-07

**Authors:** Daniel F. Tardiff, Scott A. Lacadie, Michael Rosbash

An error occurred during generation of Figure 3A for the originally published version of this article. The authors inadvertently created a splice from the original gel image, which caused a duplication of Lane 2 (HA-600), which was superimposed on the data in Lane 1 (HA-350). The left 4 lanes of the original gel image, below, correspond to Figure 3A but without this error and serve as a new Figure 3A. (The right 4 lanes of this image correspond correctly to Figure 6B.) Although regrettable and unfortunate, the authors believe that the error in producing Figure 3A had no bearing on the substance of the paper.

Relevant to this point, the authors also note that the original figure legend lacked important detail. It did not make clear that the purpose of the Figure 3A gel was only to confirm the presence of the construct and its well-characterized multiple RNA species. Given this complex pattern, gels were never used for quantitation or for any substantive conclusions. Rather, quantitative RT-PCR assays were used for all data after the Figure 3A gel with oligos as specified in the original paper’s supplemental information (Table S2). A corrected version of the figure legend appears below.

In addition, it was noted during the evaluation of Figure 3A that there was also likely a splicing of lanes in Figure S2. The authors examined the original gel image for this figure and confirmed that 2-fold dilutions of each IP (immunoprecipitated) sample were run on that gel as specified. However, only one lane was used for the final figure; hence the splice. Nonetheless, the authors have made no changes to this figure as there was no guidance about splicing of gels at the time of publication in 2006.

## Figures and Tables

**Figure 3A.**
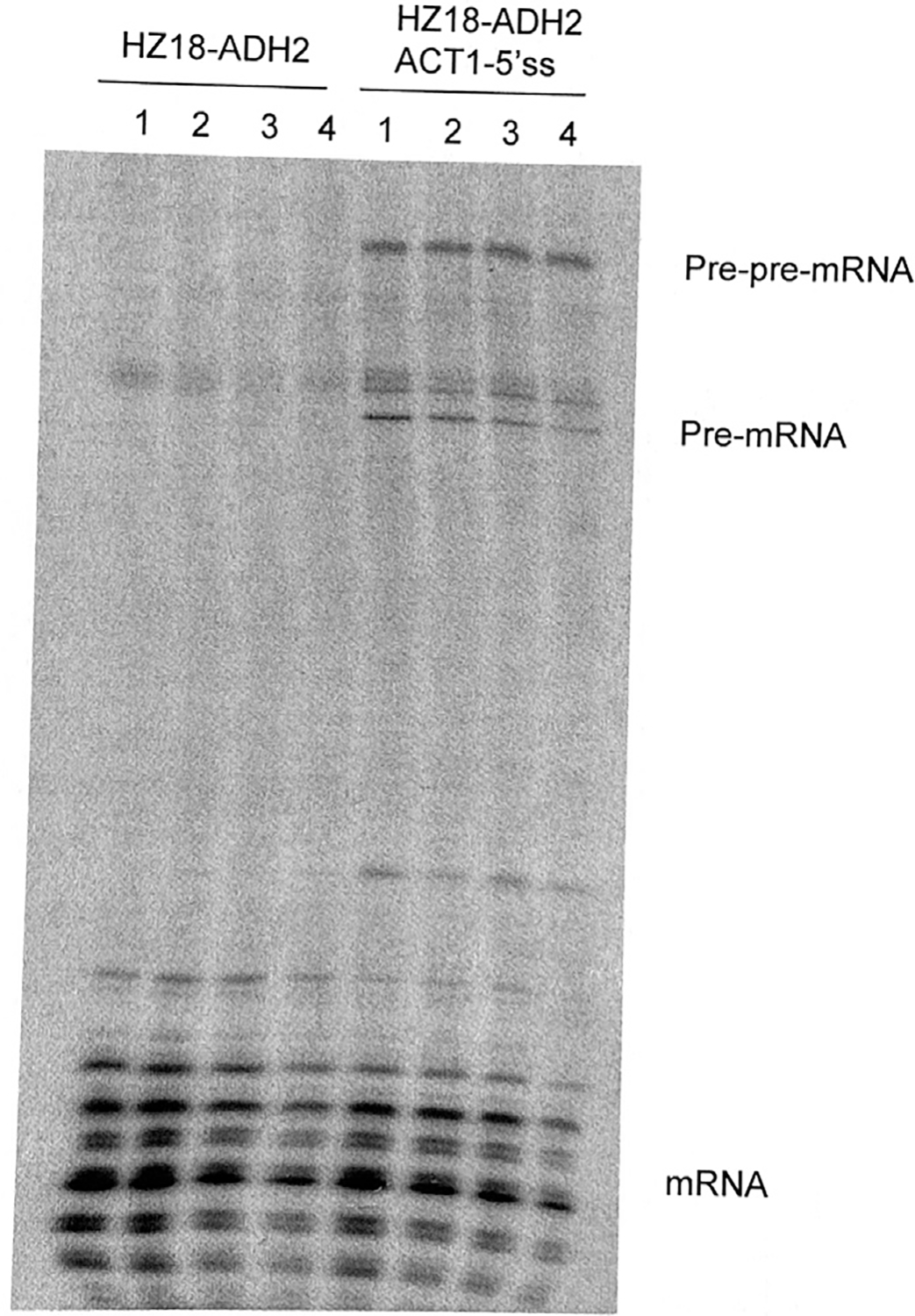
Posttranscriptional spliceosome assembly is not required for efficient splicing

